# 1829. The Impact of Social Determinants of Health on Treatment Route, Duration, and Outcomes in Bone and Joint Infections

**DOI:** 10.1093/ofid/ofad500.1658

**Published:** 2023-11-27

**Authors:** Heather Cummins, Chanah Gallagher, Julie Gray, Russell J Benefield, Laura Certain

**Affiliations:** University of Utah School of Medicine, Salt Lake City, Utah; The Medical Center of Aurora, Aurora, Colorado; University of Utah, Salt Lake City, Utah; University of Utah Health, Salt Lake City, Utah; University of Utah, Salt Lake City, Utah

## Abstract

**Background:**

The Oral versus Intravenous Antibiotics for Bone and Joint Infection (OVIVA) trial concluded that oral antibiotic therapy was noninferior to intravenous antibiotic therapy for complex orthopedic infections. Similar studies have confirmed the OVIVA trials findings. However, there is limited information regarding the impact of Social Determinants of Health (SDOH) on decisions regarding IV vs. PO treatment and treatment outcomes of bone and joint infections. This study aimed to determine how SDOH affect IV vs. PO treatment choices and overall treatment outcomes for patients with bone and joint infections.

**Methods:**

In this single-center cohort, patients discharged between 4/1/2018 and 4/1/2022 were examined. Inclusion criteria were patients who had bone and joint infections, inpatient Infectious Disease (ID) consult, discharged on IV vancomycin, age >18 years, and scheduled follow up in the University of Utah Infectious Disease Clinic. SDOH assessed included primary language, race/ethnicity, insurance, history of intravenous drug use (IVDU), and history of homelessness. The primary outcome was the number of patients switched from IV to PO antibiotics two weeks after discharge. Secondary outcomes included total duration of antibiotics, duration of follow up with ID, and rate of treatment failure.

**Results:**

Two hundred seventy-seven patients met inclusion criteria for the cohort. There was no difference with respect to history of homelessness, primary language, history of IVDU, race, ethnicity, or insurance status in patients who were switched to PO antibiotics within two weeks of discharge compared to patients maintained on IV antibiotics. With respect to secondary outcomes, individuals on Medicaid were more likely to experience treatment failure (Table 1B).

**Figure:**
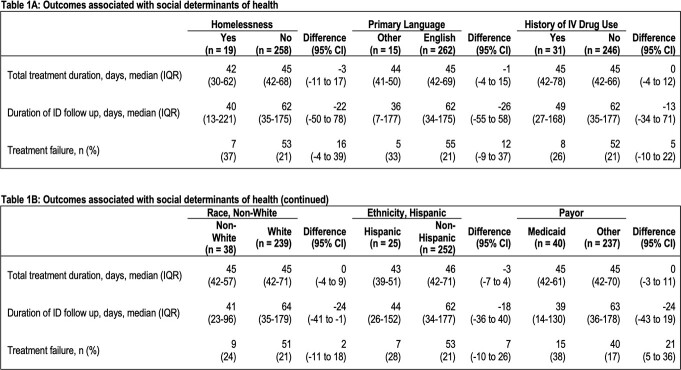

**Conclusion:**

For patients with bone and joint infections, SDOH were not associated with a switch from IV to PO antibiotics. However, patients on Medicaid experienced higher rates of treatment failure despite no difference in antibiotic route. The examined SDOH and transition to PO regimens were infrequently observed among study participants which created difficulty with drawing further conclusions. Further examination of these SDOH is important to understand their impact on treatment failure and improve patient care.

**Disclosures:**

**Russell J. Benefield, PharmD, BCPS-AQ ID**, Paratek Pharmaceuticals: Grant/Research Support

